# Supplementary material for: Use of multiple picosecond high-mass molecular dynamics simulations to predict crystallographic B-factors of folded globular proteins
Source: Heliyon. 2016 Sep 20;2(9):e00161. doi: 10.1016/j.heliyon.2016.e00161 (PMC5035356; doi:10.1016/j.heliyon.2016.e00161)
Supplement: Table S1 [file mmc1.pdf]

Supporting Information

Use of multiple picosecond high-mass molecular dynamics simulations to predict crystallographic B-factors of folded globular proteins

Yuan-Ping Pang

Computer-Aided Molecular Design Laboratory, Mayo Clinic, Rochester, MN 55905, USA

Corresponding author: Stabile 12-26, Mayo Clinic, 200 First Street SW, Rochester, MN 55905, USA; E-mail address: pang@mayo.edu; Telephone: 1-507-284-7868

Table S1A. Experimental and calculated crystallographic Cα B-factors of GB3 at 297 K over the timescale of 50 ps^{smt}.

Residue ID	PDB ID 1IGD	FF12MChm mean	FF12MChm SE	FF14SBhm mean	FF14SBhm SE	Residue ID	PDB ID 1IGD	FF12MChm mean	FF12MChm SE	FF14SBhm mean	FF14SBhm SE
6	9.37	9.0	0.9	7.0	0.4	34	8.52	4.6	0.2	4.3	0.3
7	7.26	6.2	0.4	4.6	0.2	35	5.83	2.9	0.1	2.5	0.1
8	5.67	2.6	0.1	2.0	0.0	36	8.04	3.6	0.1	3.4	0.1
9	5.25	3.1	0.1	2.5	0.1	37	8.49	5.1	0.3	4.7	0.2
10	4.92	3.0	0.1	2.6	0.1	38	6.97	5.2	0.4	4.4	0.2
11	4.74	2.9	0.2	2.5	0.1	39	7.52	4.6	0.3	4.2	0.2
12	6.55	3.9	0.5	2.8	0.2	40	10.58	6.2	0.3	5.9	0.3
13	11.08	4.8	0.5	4.1	0.3	41	10.70	8.2	0.6	6.6	0.3
14	10.70	6.6	0.5	5.5	0.5	42	8.19	7.5	0.6	6.8	0.5
15	13.87	10.7	1.1	9.1	1.1	43	12.66	8.1	0.6	7.5	0.6
16	10.83	16.4	2.6	14.2	2.1	44	10.80	4.9	0.3	4.3	0.3
17	7.46	9.1	0.9	7.2	0.5	45	13.78	6.6	0.4	6.6	0.6
18	6.02	6.3	0.5	5.7	0.5	46	10.92	9.0	0.5	6.9	0.5
19	5.14	8.4	0.5	7.6	0.4	47	6.35	5.4	0.4	4.4	0.3
20	4.57	5.3	0.2	5.1	0.2	48	5.23	4.1	0.2	3.4	0.1
21	5.79	5.2	0.3	5.2	0.3	49	5.24	4.4	0.2	3.3	0.1
22	5.73	4.6	0.2	3.8	0.2	50	5.26	4.0	0.2	3.3	0.1
23	6.63	5.9	0.4	4.7	0.2	51	6.92	4.2	0.2	4.0	0.2
24	8.78	7.0	0.5	5.3	0.3	52	8.34	6.1	0.3	5.2	0.2
25	8.12	6.1	0.4	5.7	0.5	53	14.44	9.4	0.6	7.5	0.4
26	9.17	10.0	0.9	9.5	0.9	54	8.53	6.4	0.3	5.7	0.2
27	8.29	6.1	0.3	4.9	0.4	55	7.33	3.8	0.2	3.0	0.1
28	7.92	5.5	0.3	4.2	0.2	56	5.13	3.2	0.1	2.5	0.1
29	8.29	6.1	0.4	5.5	0.4	57	4.37	2.6	0.1	2.0	0.1
30	8.62	4.9	0.3	4.8	0.4	58	5.03	3.1	0.1	2.3	0.1
31	7.76	3.6	0.2	3.3	0.2	59	5.94	3.1	0.1	2.7	0.1
32	7.49	4.0	0.2	3.7	0.2	60	6.83	3.7	0.2	3.3	0.1
33	8.72	5.0	0.2	5.0	0.4	61	9.61	4.9	0.3	5.1	0.4

SE: standard error calculated from 20 distinct, independent, unrestricted, unbiased, and isobaric–isothermal molecular dynamics simulations of GB3.

Table S1B. Experimental and calculated crystallographic Cγ B-factors of GB3 at 297 K over the timescale of 50 ps^{smt}.

Residue ID	PDB ID 1IGD	FF12MChm mean	FF12MChm SE	FF14SBhm mean	FF14SBhm SE	Residue ID	PDB ID 1IGD	FF12MChm mean	FF12MChm SE	FF14SBhm mean	FF14SBhm SE
6	15.33	23.5	2.4	10.4	0.4	35	5.99	3.6	0.1	2.9	0.1
7	13.69	15.7	1.1	10.1	0.3	36	18.85	11.2	1.0	6.0	0.4
8	7.01	3.3	0.1	2.6	0.1	37	17.37	17.4	1.4	10.0	0.5
9	8.78	14.0	1.7	5.1	0.2	38	6.92	7.8	0.7	6.9	0.5
10	6.19	5.1	0.3	4.4	0.1	40	42.35	18.2	2.3	13.0	1.0
11	8.60	8.8	0.7	6.5	0.2	41	11.80	17.2	1.5	10.7	0.5
12	12.84	18.0	2.8	6.0	0.3	42	11.29	15.5	1.5	11.7	0.9
13	17.08	14.7	1.9	11.5	1.3	44	11.81	17.5	2.0	10.4	0.9
15	34.94	23.5	2.4	19.5	2.4	45	44.17	17.9	1.7	19.0	2.5
16	16.96	42.5	6.6	32.8	5.0	47	10.98	18.7	3.6	10.0	1.1
17	8.14	20.0	2.1	14.0	0.9	48	6.51	5.4	0.2	4.6	0.1
18	14.80	19.6	2.0	9.4	0.6	49	6.81	17.8	2.1	7.9	0.3
20	8.47	17.3	1.5	9.9	0.6	50	6.65	5.2	0.2	4.2	0.2
21	8.14	11.3	0.5	9.1	0.4	51	16.64	8.4	0.5	9.5	0.5
22	9.24	12.9	1.2	7.7	0.2	52	25.01	15.3	1.7	13.3	0.8
23	10.26	14.4	1.5	7.9	0.4	54	16.48	12.9	0.6	12.0	0.6
24	28.78	23.1	1.5	13.2	1.3	55	11.18	10.1	0.9	6.4	0.5
26	11.75	24.6	3.1	18.4	1.7	56	8.87	7.5	0.3	5.2	0.2
27	10.37	10.4	0.8	8.0	0.9	57	5.42	4.4	0.2	3.2	0.1
29	39.72	13.8	1.2	9.5	0.8	58	6.18	9.0	1.0	6.0	0.2
30	10.79	12.5	1.4	9.1	0.5	59	8.12	6.8	0.2	5.4	0.2
32	8.71	9.2	0.6	7.8	0.4	60	10.01	13.4	1.8	7.9	0.4
33	20.51	15.8	1.6	11.3	1.2	61	12.85	8.4	0.8	5.7	0.4

SE: standard error calculated from 20 distinct, independent, unrestricted, unbiased, and isobaric–isothermal molecular dynamics simulations of GB3.

Table S1C. Experimental and calculated crystallographic Cα B-factors of BPTI at 297 K over the timescale of 50 ps^{smt}.

Residue	PDB ID	FF12MChm		FF14SBhm		Residue	PDB ID	FF12MChm		FF14SBhm	
ID	4PTI	mean	SE	mean	SE	ID	4PTI	mean	SE	mean	SE
1	17.63	19.6	2.0	12.4	1.2	30	7.89	3.6	0.2	2.9	0.1
2	9.21	11.3	1.0	8.5	0.7	31	9.28	3.9	0.1	3.1	0.1
3	10.58	9.6	0.9	7.6	0.4	32	10.49	4.5	0.2	3.4	0.1
4	17.01	7.8	0.9	5.4	0.3	33	10.15	4.2	0.1	3.0	0.1
5	9.61	5.7	0.3	4.0	0.2	34	7.47	5.0	0.2	3.6	0.2
6	16.06	6.1	0.5	4.2	0.2	35	8.02	4.5	0.3	2.9	0.1
7	13.10	5.3	0.3	4.9	0.3	36	8.05	5.9	0.5	3.3	0.1
8	12.90	6.0	0.3	4.9	0.2	37	7.09	10.4	0.9	5.4	0.2
9	9.50	5.3	0.3	4.1	0.1	38	6.52	8.3	0.7	5.0	0.2
10	10.93	4.2	0.2	3.5	0.1	39	11.96	10.1	0.8	6.6	0.3
11	10.34	4.0	0.2	2.8	0.1	40	15.30	8.2	0.4	5.4	0.2
12	8.45	7.6	0.5	5.3	0.2	41	12.46	4.9	0.2	4.3	0.2
13	16.32	12.7	1.2	8.7	0.6	42	7.00	4.5	0.3	4.2	0.1
14	12.04	7.8	0.5	5.4	0.3	43	6.83	2.9	0.1	2.6	0.1
15	19.55	14.1	0.8	9.5	0.8	44	10.77	3.0	0.1	2.5	0.1
16	9.98	11.4	0.4	7.3	0.5	45	5.68	3.1	0.1	2.6	0.1
17	10.09	9.6	0.5	7.8	0.8	46	8.58	6.1	0.3	4.6	0.2
18	13.44	5.8	0.4	4.2	0.2	47	7.49	9.4	0.8	5.5	0.2
19	7.64	5.3	0.4	4.3	0.2	48	6.49	7.2	0.4	5.3	0.2
20	7.78	3.1	0.1	2.6	0.1	49	6.16	6.7	0.4	5.9	0.4
21	12.57	2.9	0.1	2.7	0.1	50	10.77	6.6	0.6	4.8	0.2
22	4.10	3.1	0.1	2.6	0.1	51	12.87	3.2	0.3	2.5	0.1
23	4.91	2.6	0.1	2.1	0.1	52	8.29	4.2	0.2	3.7	0.2
24	9.31	4.5	0.3	4.1	0.2	53	11.60	6.8	0.4	5.5	0.3
25	15.09	9.8	0.6	10.8	0.7	54	9.55	7.5	0.7	4.0	0.2
26	14.48	15.6	1.5	11.3	0.6	55	10.08	6.3	0.7	3.4	0.2
27	11.73	13.5	0.8	8.3	0.4	56	16.38	10.9	1.2	10.3	0.6
28	8.56	8.5	0.5	7.5	0.4	57	28.53	26.4	4.4	26.3	2.4
29	10.25	5.2	0.4	4.4	0.3	58	45.05	50.1	6.2	54.9	7.8

SE: standard error calculated from 20 distinct, independent, unrestricted, unbiased, and isobaric–isothermal molecular dynamics simulations of BPTI.

Table S1D. Experimental and calculated crystallographic CγB-factors of BPTI at 297 K over the timescale of 50 ps^{smt}.

Residue	PDB ID	FF12MChm		FF14SBhm		Residue	PDB ID	FF12MChm		FF14SBhm	
ID	4PTI	mean	SE	mean	SE	ID	4PTI	mean	SE	mean	SE
1	13.42	17.3	1.9	10.4	1.0	26	26.23	38.1	4.0	22.1	1.4
2	11.42	16.9	1.4	12.6	1.3	29	14.01	17.3	1.0	14.9	1.5
3	34.90	18.1	2.5	18.8	1.8	31	17.66	13.4	1.0	6.2	0.2
4	13.77	8.4	0.7	6.8	0.5	32	14.64	14.8	1.0	8.0	0.2
6	20.58	18.5	1.6	9.7	0.4	33	6.68	4.5	0.2	3.2	0.1
7	29.71	10.8	1.1	8.7	1.0	34	15.81	21.3	2.9	13.9	1.6
8	27.88	11.5	0.7	12.1	0.7	35	10.18	5.3	0.4	3.8	0.1
9	10.65	7.6	0.4	6.6	0.2	39	12.04	28.1	3.1	12.5	0.6
10	9.25	6.9	1.0	5.2	0.2	41	14.28	11.3	0.6	7.2	0.5
11	23.47	11.3	2.0	5.5	0.2	42	21.62	10.0	1.3	7.7	0.6
13	15.31	20.7	1.9	13.3	0.6	43	7.77	3.0	0.1	2.5	0.1
15	32.27	37.9	3.1	25.1	3.1	44	7.36	4.4	0.3	3.0	0.1
17	9.73	20.4	1.5	18.2	1.9	45	10.01	4.4	0.3	2.9	0.1
18	11.08	12.0	0.8	6.9	0.2	46	22.12	19.3	1.7	8.9	0.4
19	14.39	17.4	2.1	10.7	0.5	49	5.98	19.7	1.5	12.4	0.9
20	11.46	4.3	0.2	2.8	0.1	50	31.36	14.7	2.1	12.0	1.3
21	5.50	5.1	0.3	4.9	0.2	52	9.44	16.0	1.3	8.4	0.8
22	10.98	4.5	0.2	3.3	0.1	53	29.13	16.9	1.9	10.6	0.8
23	6.41	4.2	0.2	3.4	0.1	54	12.27	15.9	1.6	7.0	0.4
24	22.83	10.5	0.8	6.8	0.3						

SE: standard error calculated from 20 distinct, independent, unrestricted, unbiased, and isobaric–isothermal molecular dynamics simulations of BPTI.

Table S1E. Experimental and calculated crystallographic Cα B-factors of ubiquitin at 297 K over the timescale of 50 ps^{smt}.

Residue	PDB ID	FF12MChm		FF14SBhm		Residue	PDB ID	FF12MChm		FF14SBhm	
ID	1UBQ	mean	SE	mean	SE	ID	1UBQ	mean	SE	mean	SE
1	10.38	8.0	0.6	4.9	0.3	39	14.96	8.2	0.5	6.2	0.4
2	9.07	5.8	0.3	3.5	0.2	40	10.76	6.4	0.6	4.5	0.3
3	5.07	4.6	0.2	2.8	0.1	41	3.87	4.2	0.4	3.3	0.1
4	4.68	3.7	0.1	2.6	0.1	42	6.97	4.5	0.2	3.6	0.1
5	3.87	3.2	0.1	2.6	0.1	43	3.51	4.2	0.2	3.1	0.1
6	6.12	3.7	0.1	3.0	0.1	44	5.55	4.3	0.2	3.7	0.1
7	7.48	5.5	0.3	5.2	0.4	45	4.70	4.7	0.2	4.2	0.2
8	14.15	10.9	1.7	11.0	1.1	46	7.15	16.3	1.0	12.1	0.6
9	19.24	14.6	1.3	14.8	1.0	47	11.68	17.6	1.7	13.6	0.7
10	18.74	14.9	1.0	11.4	0.9	48	8.82	9.7	1.0	7.6	0.4
11	11.91	8.5	0.5	6.1	0.6	49	7.18	6.3	0.3	5.3	0.3
12	9.85	5.8	0.3	4.7	0.2	50	7.41	5.1	0.3	4.2	0.2
13	11.84	5.8	0.4	3.9	0.2	51	11.90	6.0	0.5	4.8	0.3
14	9.63	4.8	0.2	3.7	0.1	52	16.56	5.3	0.3	4.7	0.3
15	9.03	4.1	0.3	3.2	0.1	53	11.77	7.1	0.4	6.7	0.4
16	11.50	4.7	0.4	3.6	0.2	54	9.05	4.3	0.2	3.7	0.1
17	8.85	5.0	0.5	3.5	0.2	55	9.03	4.9	0.3	3.4	0.1
18	7.08	6.6	0.5	4.4	0.3	56	8.29	5.3	0.3	3.2	0.1
19	7.07	8.7	0.9	5.1	0.2	57	9.00	8.0	0.7	4.8	0.2
20	6.28	7.3	0.5	4.4	0.2	58	7.91	9.0	0.7	4.9	0.2
21	7.70	3.9	0.2	3.1	0.2	59	8.45	8.7	0.7	5.4	0.2
22	6.01	3.9	0.2	3.0	0.1	60	13.94	9.2	0.7	6.4	0.3
23	9.92	3.5	0.2	2.8	0.1	61	11.78	6.9	0.6	4.6	0.2
24	11.81	4.2	0.2	4.0	0.3	62	15.52	10.0	1.2	6.6	0.4
25	10.96	3.7	0.1	3.8	0.2	63	11.97	8.0	0.5	4.9	0.2
26	5.53	3.2	0.1	2.9	0.1	64	10.94	6.3	0.3	4.9	0.2
27	4.14	3.0	0.1	2.6	0.1	65	6.90	5.5	0.3	4.0	0.1
28	7.74	3.5	0.1	3.1	0.1	66	3.80	4.9	0.2	3.7	0.1
29	7.90	3.6	0.2	3.0	0.1	67	3.85	5.0	0.4	3.3	0.1
30	5.58	3.8	0.2	2.8	0.1	68	4.17	3.8	0.2	2.8	0.1
31	8.67	4.7	0.2	3.5	0.1	69	3.97	3.8	0.3	3.6	0.1
32	14.01	6.2	0.3	4.9	0.2	70	6.26	5.5	0.4	4.8	0.2
33	14.00	8.7	0.7	5.0	0.2	71	16.06	7.6	0.6	6.3	0.3
34	10.07	9.6	0.7	5.5	0.2	72	25.83	8.9	0.9	6.7	0.4
35	6.29	9.0	0.5	6.0	0.2	73	30.76	14.1	1.3	13.1	1.1
36	6.07	7.3	0.4	5.2	0.2	74	35.33	27.1	3.2	15.9	1.3
37	9.18	7.6	0.4	5.4	0.3	75	36.07	61.7	7.4	44.0	4.9
38	9.08	6.3	0.3	5.0	0.3	76	36.19	97.0	10.8	82.0	6.4

SE: standard error calculated from 20 distinct, independent, unrestricted, unbiased, and isobaric–isothermal molecular dynamics simulations of ubiquitin.

Table S1F. Experimental and calculated crystallographic Cγ B-factors of ubiquitin at 297 K over the timescale of 50 ps[smt].

Residue	PDB ID	FF12MChm		FF14SBhm		Residue	PDB ID	FF12MChm		FF14SBhm	
ID	1UBQ	mean	SE	mean	SE	ID	1UBQ	mean	SE	mean	SE
1	16.29	15.4	1.2	6.4	0.3	38	10.81	12.2	0.6	12.7	1.1
2	17.01	20.2	2.3	7.2	0.4	39	31.06	15.8	1.5	11.6	1.2
3	5.58	10.2	0.4	6.2	0.2	40	14.85	14.4	1.5	10.3	1.4
4	7.97	6.5	0.4	4.5	0.2	41	3.20	4.4	0.4	3.1	0.1
5	9.13	9.0	1.1	6.6	0.2	42	21.27	13.4	1.4	6.6	0.7
6	11.12	11.6	1.1	7.6	0.8	43	6.32	8.4	0.4	5.8	0.3
7	9.17	13.4	1.8	9.5	0.4	44	7.39	14.7	1.8	8.3	0.2
8	18.88	25.8	4.1	19.6	2.5	45	5.98	7.4	0.4	7.1	0.3
9	19.70	42.0	3.8	27.8	1.8	48	14.14	23.9	2.2	18.6	1.4
11	16.69	25.3	2.6	9.9	1.3	49	15.82	15.4	1.6	9.2	0.6
12	9.63	14.5	1.7	10.4	0.4	50	7.53	7.0	0.6	5.1	0.2
13	17.08	15.9	2.5	9.0	0.5	51	26.06	18.9	2.1	10.6	0.9
14	11.66	17.0	1.6	11.5	0.7	52	25.12	10.2	1.5	8.2	0.6
15	15.79	8.7	0.8	7.2	0.6	54	9.62	11.9	1.3	9.1	0.6
16	23.33	21.0	2.2	11.7	1.4	55	11.71	10.9	1.1	6.5	0.3
17	10.54	11.3	0.7	7.1	0.6	56	7.73	9.3	1.0	4.5	0.3
18	12.65	19.1	1.8	11.0	0.8	58	11.50	14.7	1.4	6.3	0.2
19	8.16	15.3	1.4	11.1	0.7	59	6.91	7.2	0.4	5.1	0.1
21	15.32	6.0	0.4	4.9	0.3	60	22.65	21.4	1.7	17.0	2.3
22	9.65	14.7	1.6	6.6	0.2	61	13.29	14.8	1.8	8.5	0.3
23	10.90	7.1	0.3	6.4	0.3	62	26.38	25.4	3.5	16.1	1.6
24	27.76	12.8	1.4	9.3	0.8	63	16.98	17.3	1.8	8.8	1.2
25	22.31	6.1	0.9	6.3	0.3	64	24.16	16.3	1.7	10.5	0.7
26	8.12	8.1	0.3	6.5	0.2	66	3.40	15.5	1.2	8.1	0.3
27	7.45	7.0	0.9	3.4	0.2	67	9.67	10.7	0.8	6.0	0.3
29	14.94	7.7	0.5	4.8	0.4	68	9.95	9.7	1.0	7.5	0.7
30	2.78	6.5	0.2	4.5	0.1	69	7.37	7.5	0.6	5.4	0.2
31	10.76	9.1	0.5	5.3	0.2	70	8.54	19.8	2.7	11.2	0.9
32	24.33	14.5	0.7	10.4	0.9	71	19.37	17.9	1.8	15.5	2.1
33	24.00	19.6	2.0	6.2	0.4	72	31.79	14.8	1.1	11.7	1.0
34	18.75	18.2	2.3	9.7	1.1	73	30.16	42.1	3.8	34.2	3.1
36	7.36	17.7	2.7	6.9	0.3	74	38.62	51.4	6.3	22.7	2.5
37	9.27	13.3	0.7	9.3	0.6						

SE: standard error calculated from 20 distinct, independent, unrestricted, unbiased, and isobaric–isothermal molecular dynamics simulations of ubiquitin.

Table S1G. Experimental and calculated crystallographic Cα B-factors of lysozyme at 295 K over the timescale of 50 ps^{smt}.

Residue	PDB ID	FF12MChm		FF14SBhm		Residue	PDB ID	FF12MChm		FF14SBhm	
ID	4LZT	mean	SE	mean	SE	ID	4LZT	mean	SE	mean	SE
1	11.32	10.8	0.5	6.8	0.3	66	7.86	6.2	0.2	4.8	0.2
2	8.52	7.8	0.4	5.1	0.2	67	12.96	14.8	1.1	9.5	0.5
3	7.86	5.7	0.2	4.6	0.2	68	14.52	13.3	1.1	8.7	0.5
4	8.74	8.7	0.5	6.2	0.3	69	8.91	8.6	0.6	6.6	0.3
5	9.46	7.2	0.4	4.8	0.2	70	11.54	15.4	1.0	12.8	0.8
6	8.44	6.3	0.3	3.9	0.2	71	15.83	18.4	1.5	13.6	0.7
7	7.83	6.3	0.3	4.6	0.2	72	10.25	9.5	0.6	7.1	0.5
8	6.84	4.3	0.2	3.2	0.1	73	7.63	7.8	0.6	6.2	0.3
9	7.71	4.3	0.2	3.0	0.1	74	6.19	5.6	0.4	4.3	0.2
10	8.79	4.9	0.2	3.6	0.2	75	6.16	6.2	0.5	5.2	0.3
11	8.45	5.2	0.2	4.2	0.2	76	5.84	6.7	0.5	4.7	0.2
12	7.70	4.5	0.1	3.2	0.1	77	6.35	8.0	0.4	6.2	0.3
13	9.64	5.8	0.3	4.0	0.2	78	7.13	7.2	0.4	5.0	0.2
14	10.42	7.7	0.5	5.6	0.3	79	7.37	6.8	0.4	4.5	0.2
15	11.72	8.6	0.8	5.7	0.3	80	6.23	4.0	0.2	3.0	0.1
16	13.62	9.3	0.7	7.3	0.5	81	7.42	7.1	0.4	5.8	0.4
17	9.59	7.3	0.5	5.4	0.2	82	7.49	10.2	1.2	6.2	0.5
18	9.89	7.5	0.4	6.4	0.3	83	7.35	8.5	1.1	4.0	0.2
19	8.60	7.2	0.3	6.5	0.3	84	8.22	7.0	0.6	4.5	0.2
20	8.45	6.7	0.3	5.4	0.2	85	13.32	8.8	0.6	6.1	0.3
21	9.39	8.9	0.6	6.2	0.2	86	13.52	9.0	0.6	6.3	0.3
22	9.97	12.0	0.9	8.5	0.3	87	12.57	7.0	0.5	6.0	0.3
23	8.44	6.5	0.4	5.5	0.3	88	8.57	4.6	0.3	4.2	0.2
24	9.19	6.1	0.3	4.8	0.2	89	10.16	4.5	0.3	3.7	0.2
25	8.38	4.2	0.1	3.2	0.1	90	8.21	4.5	0.2	4.2	0.2
26	8.78	4.4	0.2	3.4	0.1	91	6.50	4.1	0.1	3.6	0.1
27	7.49	4.0	0.2	2.7	0.1	92	7.88	4.2	0.2	3.4	0.2
28	6.96	4.3	0.2	3.3	0.1	93	8.16	4.5	0.2	3.5	0.2
29	6.86	3.2	0.1	2.7	0.1	94	6.27	3.7	0.2	3.0	0.1
30	7.58	3.2	0.1	2.5	0.1	95	6.93	4.4	0.2	4.0	0.2
31	7.56	4.3	0.2	3.4	0.1	96	8.22	4.9	0.2	4.6	0.3
32	6.66	4.5	0.2	3.4	0.1	97	10.17	5.2	0.4	4.6	0.2
33	7.18	4.6	0.3	3.3	0.1	98	9.90	5.3	0.4	4.1	0.2
34	7.91	4.6	0.2	3.6	0.1	99	14.59	5.9	0.6	4.5	0.2
35	6.84	3.8	0.1	3.2	0.1	100	19.08	8.2	1.0	6.4	0.3
36	8.04	5.1	0.2	3.9	0.1	101	16.35	9.4	0.7	7.4	0.4
37	9.17	6.8	0.5	4.1	0.1	102	26.69	18.3	4.9	11.7	0.6
38	7.56	5.3	0.3	3.3	0.1	103	18.77	10.7	1.6	7.5	0.5
39	8.13	5.0	0.2	3.8	0.2	104	17.38	12.7	1.3	10.6	0.7
40	7.32	5.0	0.3	3.5	0.2	105	9.91	8.0	0.6	6.1	0.5
41	8.55	6.4	0.4	4.3	0.2	106	16.70	9.3	0.7	6.5	0.5
42	7.95	5.7	0.2	4.4	0.2	107	13.93	10.1	0.6	8.1	0.7
43	8.35	5.7	0.3	4.3	0.2	108	9.30	6.4	0.5	5.6	0.4
44	8.61	6.4	0.4	5.6	0.3	109	10.67	9.0	0.8	6.9	0.4
45	8.37	8.9	0.7	8.0	0.5	110	10.52	8.4	0.7	6.8	0.3
46	9.47	10.8	0.9	9.6	0.5	111	9.74	6.7	0.5	4.8	0.2
47	10.18	22.3	2.5	18.5	1.4	112	11.51	8.0	0.7	5.4	0.2
48	12.81	18.3	1.6	14.5	1.1	113	12.38	10.7	0.9	7.6	0.3
49	14.58	13.6	1.1	12.5	0.8	114	13.70	8.4	0.2	5.9	0.2
50	7.85	6.0	0.5	4.9	0.3	115	10.90	6.1	0.3	4.0	0.1
51	6.11	3.4	0.2	3.0	0.1	116	12.73	9.2	0.7	6.2	0.4
52	5.99	3.0	0.1	2.7	0.1	117	28.58	22.3	1.7	15.8	1.1
53	5.88	2.7	0.1	2.3	0.1	118	18.53	14.4	1.3	8.2	0.4
54	6.70	4.2	0.2	3.2	0.1	119	22.82	13.4	0.9	8.7	0.6
55	6.60	3.8	0.1	2.9	0.1	120	16.41	7.5	0.4	5.6	0.2
56	6.46	4.3	0.2	3.7	0.2	121	17.92	8.6	0.5	5.8	0.3
57	5.69	3.6	0.2	3.1	0.1	122	17.95	8.3	0.5	6.0	0.3
58	6.08	3.2	0.2	3.0	0.1	123	15.88	8.1	0.5	5.9	0.3
59	6.66	3.3	0.1	3.1	0.1	124	13.70	8.3	0.5	5.6	0.2

60	6.45	3.2	0.2	2.5	0.1	125	17.70	10.6	0.4	7.2	0.3
61	6.87	4.1	0.2	3.8	0.2	126	24.68	16.5	1.1	11.4	0.5
62	8.61	5.2	0.3	4.3	0.2	127	17.40	13.2	1.1	6.5	0.3
63	5.54	3.7	0.2	3.0	0.1	128	24.46	21.9	2.1	12.8	1.0
64	7.21	4.0	0.1	3.0	0.1	129	31.79	22.2	2.4	14.0	1.3
65	8.33	5.5	0.2	3.8	0.1						

SE: standard error calculated from 20 distinct, independent, unrestricted, unbiased, and isobaric–isothermal molecular dynamics simulations of lysozyme.

Table S1H. Experimental and calculated crystallographic Cγ B-factors of lysozyme at 295 K over the timescale of 50 ps[smt].

Residue	PDB ID	FF12MChm		FF14SBhm		Residue	PDB ID	FF12MChm		FF14SBhm	
ID	4LZT	mean	SE	mean	SE	ID	4LZT	mean	SE	mean	SE
1	12.35	21.8	2.4	10.0	1.5	63	7.45	5.0	0.3	4.0	0.2
2	14.09	25.1	2.7	9.6	0.3	65	15.36	15.2	1.2	9.0	0.5
3	7.97	6.7	0.4	4.7	0.1	66	7.29	7.5	0.4	5.2	0.2
5	20.89	13.1	1.2	7.1	0.3	68	22.58	28.4	3.5	12.2	0.9
7	9.87	18.0	1.5	9.7	0.6	69	8.97	11.6	0.6	8.8	0.3
8	7.83	6.4	0.3	4.2	0.1	70	18.37	23.5	1.3	21.1	1.1
12	7.50	7.1	0.4	4.2	0.2	73	9.24	19.7	1.4	12.3	1.2
13	18.22	12.6	0.7	6.6	0.3	74	7.80	7.2	0.4	5.6	0.2
14	15.89	16.2	1.2	8.7	0.6	75	7.30	13.5	1.5	7.8	0.4
15	15.32	9.4	0.9	7.6	0.5	77	9.07	17.2	1.0	13.1	0.7
17	10.87	8.8	0.5	6.2	0.3	78	12.43	26.2	4.4	10.8	0.6
18	13.04	11.7	0.7	13.9	1.3	79	11.19	12.1	0.7	10.0	0.4
19	8.80	15.3	1.0	16.4	1.3	83	8.87	7.5	0.5	4.3	0.2
20	8.92	7.6	0.3	5.9	0.3	84	8.75	6.7	0.4	5.5	0.4
21	13.48	16.9	1.2	10.3	0.9	87	27.18	12.2	0.8	11.2	0.9
23	9.19	8.3	0.8	7.6	0.5	88	14.78	10.0	0.8	8.9	0.4
25	10.31	7.8	0.7	4.5	0.1	89	14.81	12.1	1.7	8.2	0.3
27	9.25	6.5	0.4	4.4	0.3	92	15.53	10.2	1.2	7.0	0.3
28	7.21	4.7	0.2	3.9	0.1	93	13.45	15.1	2.2	7.4	0.4
29	9.67	7.1	0.6	5.3	0.2	96	9.84	9.4	1.3	5.9	0.7
33	11.22	10.6	0.7	6.2	0.2	97	17.00	15.4	1.4	8.2	0.5
34	9.38	5.7	0.2	4.6	0.1	98	14.36	10.3	0.7	6.9	0.1
35	8.52	5.4	0.2	4.7	0.2	99	22.69	11.1	1.3	7.7	0.3
37	19.55	17.2	1.3	9.2	1.2	101	13.06	18.3	1.7	9.6	0.7
38	8.29	6.5	0.6	3.5	0.1	103	24.15	17.2	2.6	10.7	0.6
39	12.07	8.9	0.5	6.2	0.4	105	9.92	14.4	1.2	10.3	1.1
40	10.96	9.4	0.6	6.6	0.3	106	31.54	18.6	1.7	10.4	0.5
41	19.79	16.1	1.3	13.0	2.0	108	8.85	6.7	0.4	6.6	0.4
43	14.25	14.6	1.7	6.9	0.2	109	16.69	25.4	2.3	16.8	1.6
44	13.80	12.5	1.5	11.4	0.5	111	10.08	8.1	0.6	5.6	0.3
45	15.18	20.7	2.5	13.5	0.8	112	39.74	15.7	1.4	9.2	0.4
46	11.42	10.6	0.6	9.4	0.4	113	24.29	22.0	3.1	16.2	0.7
47	13.42	51.9	4.0	33.8	2.4	114	16.26	19.8	1.3	9.9	0.4
48	14.97	17.8	1.8	12.7	1.0	116	16.02	18.6	1.5	9.4	0.8
51	8.01	7.1	0.5	4.6	0.2	118	23.00	25.1	2.3	12.4	0.4
52	8.99	6.4	0.6	5.6	0.2	119	44.67	19.2	1.6	14.2	1.1
53	6.53	3.6	0.1	2.7	0.1	120	17.87	14.2	1.5	8.9	0.3
55	11.36	9.1	0.9	5.3	0.2	121	35.69	19.0	1.9	10.4	0.6
56	10.45	12.8	0.9	7.7	0.2	123	16.42	8.4	0.5	6.5	0.3
57	7.02	4.6	0.2	3.9	0.1	124	20.24	13.9	1.3	9.8	0.4
58	8.11	7.8	0.7	6.0	0.2	125	24.75	20.4	1.1	11.8	0.7
59	7.70	5.3	0.3	4.4	0.2	128	37.18	49.7	6.1	25.0	1.9
61	11.91	10.1	0.9	7.4	0.4	129	18.26	19.7	2.1	10.9	0.7
62	12.45	12.6	1.1	9.4	0.9						

SE: standard error calculated from 20 distinct, independent, unrestricted, unbiased, and isobaric–isothermal molecular dynamics simulations of lysozyme.
